# Open source software for the analysis of corneal deformation parameters on the images from the Corvis tonometer

**DOI:** 10.1186/s12938-015-0027-3

**Published:** 2015-04-11

**Authors:** Robert Koprowski

**Affiliations:** Department of Biomedical Computer Systems, University of Silesia, Faculty of Computer Science and Materials Science, Institute of Computer Science, ul. Będzińska 39, Sosnowiec, 41-200 Poland

**Keywords:** Cornea vibration, Cornea deformation, Tonometer, Corvis, Corvis ST, Oculus Optikgeräte GmbH, Wetzlar, Eye, Biomechanics, Image processing, Scheimpflug camera

## Abstract

**Background:**

The software supplied with the Corvis tonometer (which is designed to measure intraocular pressure with the use of the air-puff method) is limited to providing basic numerical data. These data relate to the values of the measured intraocular pressure and, for example, applanation amplitudes. However, on the basis of a sequence of images obtained from the Corvis tonometer, it is possible to obtain much more information which is not available in its original software. This will be presented in this paper.

**Material and method:**

The proposed software has been tested on 1400 images from the Corvis tonometer. The number of analysed 2D images (with a resolution of 200 × 576 pixels) in a sequence is arbitrary. However, in typical cases there are 140 images. The proposed software has been written in Matlab (Version 7.11.0.584, R2010b). The methods of image analysis and processing and in particular edge detection and the fast Fourier transform have been applied.

**Results and discussion:**

The software allows for fully automatic (1) acquisition of 12 new parameters previously unavailable in the original software of the Corvis tonometer. It also enables off-line (2) manual and (3) automatic browsing of images in a sequence; 3D graph visualization of: (4) the corneal deformation and (5) eyeball response; 6) change of the colour palette; (7) filtration and (8) visualization of selected measured values on individual 2D images. In addition, the proposed software enables (9) to save the obtained results for further analysis and processing.

**Conclusions:**

The dedicated software described in this paper enables to obtain additional new features of corneal deformations during intraocular pressure measurement. The software can be applied in the diagnosis of corneal deformation vibrations, glaucoma diagnosis, evaluation of measurement repeatability and others. The software has no licensing restrictions and can be used both commercially and non-commercially without any limitations.

**Electronic supplementary material:**

The online version of this article (doi:10.1186/s12938-015-0027-3) contains supplementary material, which is available to authorized users.

## Background

The Corvis tonometer allows for automatic measurement of intraocular pressure (IOP) by means of the air-puff method [1]. This is one of the methods for measuring pressure in the eye which provides much more information than the known techniques used, for example, in the Goldman tonometer or Ocular Response Analyzer (ORA). By using the Ultra-High-Speed Scheimpflug camera [1], the Corvis tonometer also allows for the observation and measurement of other additional biomechanical parameters based on image analysis of dynamic corneal deformations and eyeball responses. Currently, most of the works in the field of ophthalmology [2-4] or the mechanics of the eye [5-7] are related to the comparison of the results of eye pressure measurements for different tonometers and for various ophthalmic diseases starting with glaucoma [8] and ending with the population of people living in a particular region of the world [9]. For example, the authors in paper [10] analysed patients with high myopia and moderate myopic astigmatism, who were assessed as eligible for surgery and underwent small incision lenticule extraction. In paper [11], virgin and post-photorefractive keratectomy (PRK) eyes were analysed and then the results between these two groups were compared. A similar comparison was done in publications [12,13] for keratoconic and normal eyes, in [14] for three corneal transplantation techniques, or in [15] after corneal refractive surgery. There are also interesting results presented in papers [16-20] which also relate to the comparison of different groups of patients. For example, a very interesting attempt to compare the theoretical simulation results with the actual data was presented in paper [20]. Numerical results in this publication ([20]) showed that during the air puff deformation, there would be vibrations together with the corneal deformation, and the damping viscoelastic corneal response was able to reduce the vibration amplitude.

The second area (mechanics of the eye) involves modelling of corneal deformations. It is presented in a smaller number of works that still try to explain all complex phenomena occurring in the eye in dynamic states [20]. The software supplied by Oculus Optikgeräte GmbH in addition to intraocular pressure also measures other additional biomechanical parameters such as the location and amplitudes of two applanations. Moreover, the original software (version V 1.00r24) shows the graphs of deformation amplitude and deformation rate over time [21]. Although the new original software (ver. 2.00 V) enables to show additional graphs, e.g. the corneal deformation amplitude separated from the eyeball response amplitude, their quality and repeatability still leave a lot to be desired - for example, negative deformations are obtained or values are off the scale. The complete 3D visualization of corneal deformations over time is not possible.

On the other hand, it is possible to use the methods of image analysis and processing to a sequence of corneal deformation and eyeball response images. The corneal contours, both internal and external, are analysed in subsequent images of the sequence. The result is not only the complete 3D visualization of corneal deformation but also the possibility of automatic measurement of other additional parameters. These are new parameters that are measured irrespective of the software of the Corvis tonometer. The various parameters and methods of their measurement have been described in five publications by Koprowski R. [21-25] and are not cited here. The exact list of these publications and additional measurable parameters that are not available in the original software of the Corvis tonometer are shown in Table 1.

**Table 1:** **Summary of known parameters available in the original software of the Corvis tonometer and new parameters measured in the described software**

**Parameter**	**Corvis software V 1.00r24**	**Manuscript Koprowski [** 21 **]**	**Manuscript Koprowski [** 22 **]**	**Manuscript Koprowski [** 23 **]**	**Manuscript Koprowski [** 24 **]**	**Manuscript Koprowski [** 25 **]**	**Software described in this paper**
Intraocular pressure (IOP)	*						
Pachymetry	*						
Applanation 1 time	*				*		*
Applanation 1 length	*				*		*
Applanation 1 velocity	*						
Applanation 2 time	*				*		*
Applanation 2 length	*				*		*
Applanation 2 velocity	*						
Highest concavity time	*				*		*
Peak distance	*						*
Radius deformation	*				*		*
Maximum deformation amplitude (eye ball and cornea)	*				*		*
Corneal length changes		*			*		*
Ratio - amplitude changes/corneal deformation length		*			*		*
Ratio - corneal reaction/corneal static position		*			*		*
Maximum amplitude of the eyeball reaction		*					*
Maximum amplitude for the frequency >100 Hz		*				*	*
Time cornea deformations >100 Hz		*				*	*
Distinction between the left and right eye			*	*			
Asymmetry in the work of muscles - left or right eye			*	*			*
Absolute of the cornea reaction			*				*
Scleral reaction asymmetry			*				*
Median filtering of deformation		*	*	*	*	*	*
3D visualization of corneal deformation		*	*	*	*		*
3D visualization of the eyeball response		*	*	*	*		*
Repeatability analysis of corneal deformation f > 100 Hz						*	

In summary, the proposed software enables automatic and reproducible measurement of additional biomechanical parameters unavailable in the original software of the Corvis tonometer. In particular, the proposed software has the following advantages:

- fully automatic measurement of biomechanical parameters,
- measurement of: applanation 1 and 2, highest concavity time, peak distance, radius deformation, maximum deformation amplitude, and their 2D visualization, also available in the original software of the Corvis tonometer,
- measurement of: corneal length changes, ratio - amplitude changes/corneal deformation length, maximum amplitude of the eyeball reaction, maximum amplitude for the frequency >100 Hz, distinction between the left and right eye, asymmetry in the work of muscles (left or right eye) and the absolute of the cornea reaction – unavailable in the original software of the Corvis tonometer,
- 3D visualization of: corneal deformation, eyeball response, corneal steady state (constant component), corneal vibration and the Fourier transform (FFT) of corneal vibration - unavailable in the original software of the Corvis tonometer),
- full reproducibility of the measured parameters and the correct operation of the program for all the analysed images,
- full, free of charge, availability of the source codes of the program that can be used without licensing restrictions,
- lack of functional limitations associated with the development of the proposed program with new elements.

A detailed description of the proposed software is provided in the following sections while the description of the methods of measurement of individual parameters and their diagnostic significance have been described in detail in the aforementioned previous works of the author [21-25].

### Material

The proposed software has been tested for 1400 images acquired from the Corvis tonometer. The number of analysed 2D images (with a resolution of 200 × 576 pixels) in a sequence is arbitrary and in the test cases there were 140 images recorded every 231 μs. The sequence of images was acquired in *.jpg image format and *.avi video format. There was no effect of the type of eye disease (the impact of glaucoma, corneal thickness, cornea transplants, complications or detachment) on the analysis quality and presented software malfunction.

The described new software has been written in Matlab (version 7.11.0.584, R2010b) in Windows 7 Professional, 64-bit. Tests of the operation speed of individual modules were carried out on a PC with the Intel Core i7-4960X CPU @ 3.60 GHz. The components from Matlab enabling the creation of a graphical user interface (GUI), methods of image analysis and processing and in particular edge detection and the fast Fourier transform from Image Processing toolbox (version 7.1 R2010b) have been applied.

## Implementation

The software has a modular structure that enables to perform easy and quick modifications and additions to the program according to individual requirements. It consists in particular of two main files *GUI_Corvis.m* and *GUI_Corvis_fun.m*. The first one (*GUI_Corvis.m*) is responsible for visualization of the graphical user interface, i.e.: the distribution of the buttons, sliders and menu items. The other file, function *GUI_Corvis_fun.m*, is responsible for the usage. Among other things, this function is responsible for the slider operation, reaction to pressing the buttons etc. In addition to these two functions, there are three others responsible for the correct detection of the corneal contour (*Corvis_read_fun.m*), analysis of all biomechanical parameters (*GUI_Corvis_anal.m*) and conversion of file names required for their correct reading (*GUI_Corvis_corr_st*). A summary of all these five application files with their functionality is shown in Table 2 (please see Additional file 1 - GUI_Corvis.zip). Details of the source code due to its large volume (several hundred lines) included in each file will not be discussed here. Both new as well as known algorithms and functions have been applied there, e.g.: *uicontrol, imshow, imread, figure* and others.

**Table 2:** **Summary of 5 files used in applications and their purpose**

**File name**	**Purpose**
*GUI_Corvis.m*	User graphical interface – run as the first one
*GUI_Corvis_fun.m*	The function to handle individual buttons and slider
*Corvis_read_fun.m*	The function to convert a single 2D image into two waveforms of the contour - inner and outer corneal contours
*GUI_Corvis_anal.m*	The function designed to calculate all corneal biomechanical parameters based on the 3D data of cornea edges for the subsequent images in a sequence
*GUI_Corvis_corr_st*	The function to convert input file names

**Figure 1:**
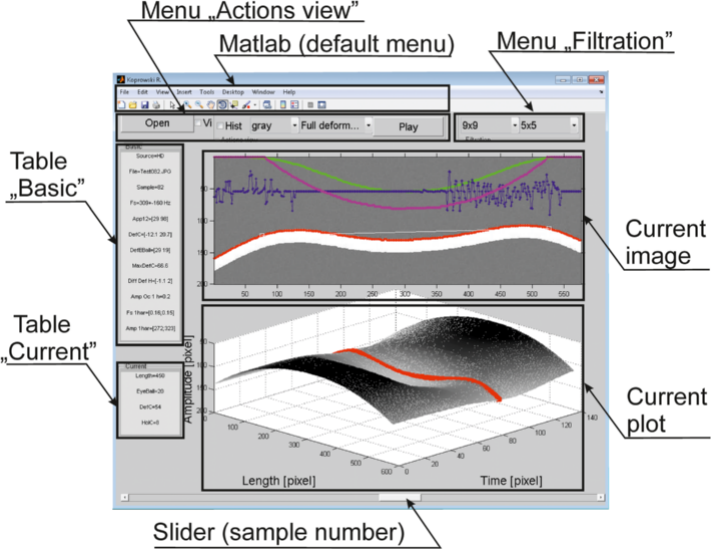
Main window in the program. The division of the main window into several sectors is clearly visible. The sectors form the menu items - on the left and on top, as well as items related to visualization - on the right at the bottom and in the middle of the window.

The main window of the program created based on all of the aforementioned files (in particular the first one, i.e.: *GUI_Corvis.m*) is shown in Figure 1. It consists of several areas:

**Menu “Action view”** – the menu designed to open an image sequence and manage visualization of results. It consists of the “Open” button intended to open an image sequence by pointing to any single *.jpg image. The checkbox “Vi” is associated with visualization of results during processing (only for new unopened image sequences). The “Hist” box makes it possible to enable or disable automatic histogram equalization while viewing and analysing an image sequence. Another element on the right-hand side is the submenu for changing the artificial colour palette on images. The user can choose from among 4 possibilities: (1) “gray” - the gray levels; (2) “jet” -ranges from blue to red; (3) “hsv” - the hue component of the hue-saturation-value colour model; (4) “hot” - varies smoothly from black through shades of red, orange, and yellow, then white. Another element is the submenu for visualization of different waveforms of the analysed image sequences. These are: (1) “Full deformation” allowing for 3D visualization of complete deformation of the cornea and the eyeball; (2) “Eye Ball” allowing for 3D visualization of the eyeball response; (3) “Cornea” - 3D visualization of the corneal deformation; (4) “Constant” - 3D visualization of the constant component - the cornea before deformation; (5) “Oscylation” - 3D graph of corneal vibrations above 100 Hz; (6) “Diff static” - 3D graph of the difference in the corneal deformation relative to the stable state; (7) “FFT” - graph of the fast Fourier transform amplitude for each image point; (8) “Graph 2D” - 3 2D graphs related to the reaction of the cornea, eyeball and applanation point. The last component of this menu is the “Play” button which allows for automatic viewing of subsequent 2D images in a sequence every 0.1 second.

**Menu “Filtration”** – the menu designed to filter the graphs of: (1) deformation of the cornea and the eyeball as well as (2) corneal deformation for the frequency > 100 Hz using a median filter. In both cases, the median filter may take the following mask sizes: “none”, “3 × 3”, “5 × 5”, “9 × 9”, “11 × 11”, “23 × 23” pixels. Filtration effects are directly reflected both in the data and 3D graphs.

**Current image** – the area of the currently analysed 2D image of the cornea and waveforms of the calculated results. The following colours were used to mark: (1) green – corneal deformation; (2) light blue - static corneal deformation [24]; (3) blue – corneal deformation for frequencies above 100 Hz; (4) red - detected outer corneal contour.

**Current plot** – area of 3D or 2D graphs depending on the choice in the “Action view” menu. The colour of the 3D graph is dependent on the selected colour palette. Red in the 3D graph highlights the waveform corresponding to the currently displayed 2D image above (“current image” area). In the case of 2D visualization (the choice of “Graph 2D” in the menu “Action view”), there are three 2D graphs: (1) red – corneal response; (2) blue – eyeball response, and (3) green – applanation points. In addition, a blue vertical line indicates the place of calculations resulting from the 2D image displayed in the upper graph.

**Slider “sample number”** - the slider available to the user which enables to watch subsequent 2D images in a sequence and moved automatically after pressing the “Play” button. The slider operates in the range from 0 to 139 of the viewed image.

**Figure 2:**
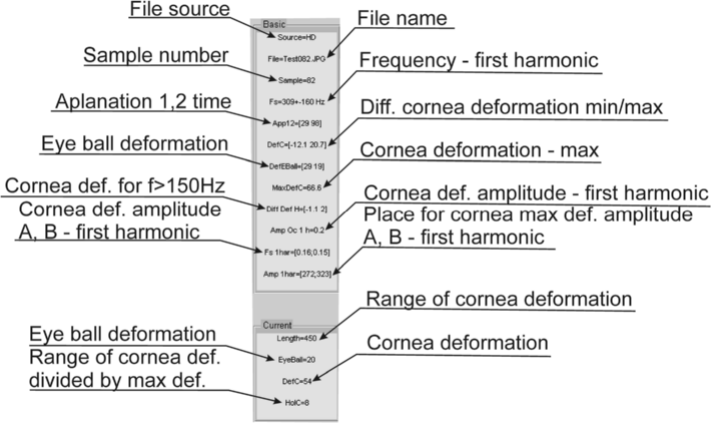
Elements of information tables - “Basic” and “Current”. The individual elements in the table define the parameters measured for the selected patient. The first table is related to the calculations carried out for the entire sequence of 2D images. The other table refers to the calculations carried out for a single 2D image - currently displayed.

**Table “Basic”** – the area of data visualization related to the currently analysed patient (image sequence). A detailed description and interpretation of the individual values are shown in Figure 2. Due to the different image sources, various types of the Corvis tonometers, “pixels” were adopted everywhere as the basic data conversion (unless stated otherwise). The user can freely convert a given unit by using simple operations. In the case of performing filtration with any filter in the “Filtration” menu, all the data will be recalculated.

**Table “Current” –** the area of data visualization directly related to the currently viewed 2D image in Current image. Measuring points are also marked with a blue line when selecting “2D Graph” in the menu “Action view”. For the same reasons as in the Table “Basic” all values are in pixels.

**Matlab (default menu)** - Matlab default menu enabling to record, rotate or describe the image. In addition, it enables to print and display the colour palette, zoom in or out the image, and read the values of manually indicated pixels.

**Figure 3:**
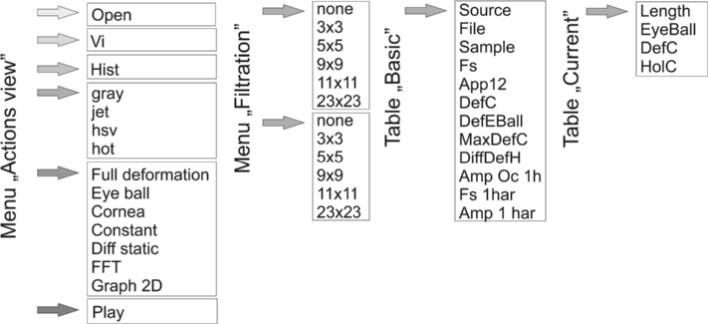
Block diagram showing possibilities of developing individual menu items of the presented software. The user has access to all program features and the results obtained are directly visible in the subsequent program windows.

The block diagram of the discussed areas of the main application window is shown in Figure 3**.** Below there are comments on the use and functionality of the proposed software.

## Results and discussion

Management of the proposed software is intuitive. After pressing the Open button, the user points to any file in the sequence of *.jpg images acquired from the Corvis tonometer. At this stage, the existence of the *.mat file in the same location on the disk is pre-verified. In the case of its absence, the analysis of the outer corneal edge and record of the obtained results to the *.mat file begin, so that no calculations have to be performed again when reviewing the same patient. During the first analysis, the preview of 2D images and partial results can be enabled or disabled (“Vi” checkbox). It is also possible to implement histogram equalization (“Hist” checkbox). However, this slows down the analysis. After analysing the image sequence or reading ready data from the *.mat file (the program does it automatically), the results appear in the tables “Basic” and “Current”. It is now possible to view the results by selecting in the submenu (Menu “Action view”) one of the options: “Full deformation”, “Eye ball”, “Cornea”, “Constant”, “Oscylation”, “Diff static”, “FFT” or “Graph 2D”. 3D graphs (or 2D graphs when selecting “Graph 2D”) corresponding to the choice appear in the “Current plot” window. On these graphs, the coloured line (whose colour depends on the type of the viewed graph) marks the place resulting from calculations for the currently displayed image in the “Current image” area. With the slider placed at the bottom of the application, it is possible to view and analyse each image in the sequence. In the presence of artefacts in images caused by, for example, improperly detected contours of the cornea or noise in the image, median filtering for one of the available masks can be performed - field of “Filtration” Menu. Examples of image sequence analysis results are shown in Table 3 for 140 images in the sequence archived every 231 μs.

**Table 3:** **Sample numerical results obtained from the presented software (in brackets they are given after unit conversion for the size 0.15 mm/pixel and time 231 μm/pixel)**

**Measured parameter**	**Value**
Data source origin	Hard drive
Source File	Test082.jpg
Image number	82
Frequency of the first harmonic	309 ± 160 Hz
Applanation time	29 and 96 pixels (6.7 ms, 22.2 ms)
Difference in corneal deformation	Minimum −12.1 pixels (1.8 mm) and maximum 20.7 pixels (3.1 mm)
Amplitude of the eyeball response	29 and 19 pixels (4.3 mm, 2.9 mm)
Maximum corneal deformation	66.6 pixels (10 mm)
Maximum corneal deformation for the frequency > 100 Hz	−1.1 2 pixels (−1.7 mm, 0.3 mm)
Amplitude of the first harmonic	0.2 pixel (0.03 mm)
Amplitude of the first harmonic measured at the highest points of curvature	0.16 and 0.15 pixel (24 μm, 22 μm)
Location of points with the greatest curvature during deflection over time	272 and 323 pixels (63.1 ms, 74.6 ms)

**Figure 4:**
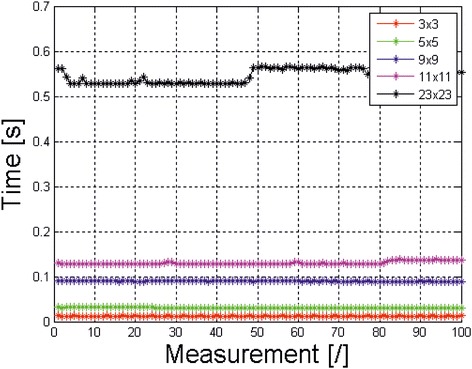
Waveform of the filtration rate with an averaging filter for different mask sizes for 100 tests. The average values of median filtering rate for the Intel Core i7-4960X are: for the mask sized 3 × 3 pixels - 12 ± 0.4 ms, 5 × 5 pixels - 31 ± 0.7 ms, 9 × 9 pixels - 89 ± 0.8 ms, 11 × 11 pixels - 0.13 ± 0.003 s, 23 × 23 pixels - 0.54 ± 0.015 s.

Time analysis of individual modules for Windows 7 Professional, 64-bit with the Intel Core i7-4960X CPU @ 3.60 GHz and HDD 3.5″ SATA III 2 TB) is presented in Table 4. The analysis rate is mainly affected by the processor speed. When reading data the speed of access to the hard drive is also important. For SSD (e.g. SAMSUNG 840 EVO MZ-7TE500BW 2.5″ 500 GB SSD), the total analysis time can be reduced even 10-fold. The calculation time is to a lesser extent influenced by the type of Matlab - in this case, Version 7.11.0.584, R2010b and the type of operating system (Windows/Linux). Figure 4 shows examples of rate changes in the median filtering of a data sequence for different mask sizes for 100 tests (500 GB 2.5″ SSD, Intel Core i7-4960X). According to the graph, the filtration times were as follows: for the mask sized 3 × 3 pixels it was 12 ± 0.4 ms, 5 × 5 pixels - 31 ± 0.7 ms, 9 × 9 pixels - 89 ± 0.8 ms, 11 × 11 pixels - 0.13 ± 0.003 s, and 23 × 23 pixels - 0.54 ± 0.015 s.

**Table 4:** **Time analysis of individual software modules**

**Measurement range**	**Time**
Reading a sequence of images (*.mat file)	From 1 to 2 seconds depending on the speed of access to the hard drive
Time of viewing the entire sequence of images (“Play” button)	37 seconds
Median filtering of the full image sequence with a mask sized 23 × 23 pixels	<1 second
Other operations of viewing data	<1 second

**Figure 5:**
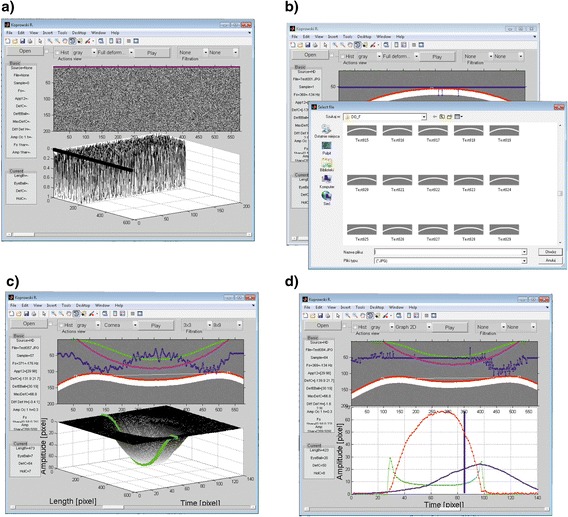
Selected various steps of software operation: **a**) the main window just after starting without downloaded data, **b**) the stage of indicating any file from a sequence of *.jpg images, **c**) corneal deformation analysis, **d**) 2D graphs for the currently analysed 2D image.

The presented software is currently used in a number of ophthalmic clinics in the world providing new diagnostic quality which is not available in the original software of the Corvis tonometer [21-25]. In particular it enables 3D visualization of the corneal deformation and eyeball response. Ophthalmologists also have new reproducible quantitative information on the frequency of the first harmonic, vibration, difference in the corneal deformation (minimum and maximum amplitude), eyeball response (thus it is possible to detect automatically which eyeball is tested - left/right) and current measurement of the corneal deformation and eyeball response dependent on the viewed 2D image. Practical clinical usefulness of the measured features is described in detail in [21-25]. In particular, the presented software can be used to assess the reproducibility of vibrations or other corneal deformations. As shown in paper [25], the vibration amplitude for subsequent measurements of the same patient is reduced exponentially. In paper [1] describing clinical applications of the Corvis tonometer, and in practice, the analysis of patients with keratoconus, their strong relationship with vibrations can be demonstrated. With the presented program, it will be possible to measure them quantitatively and not qualitatively. In addition, the proposed software will enable to analyse the impact of other ophthalmic diseases on biomechanical parameters. The analysis of a sequence of patients and their statistical analysis, owing to the automatic data record on the disk, will be also possible, which was previously unavailable in the original software. For all the analysed cases there is full reproducibility of the results obtained. Moreover, some biomechanical characteristics also present in the original software of the Corvis tonometer can be measured. For these selected features (see Table 1 - for example the first and second applanation) there is measurement consistency of 96%. The difference of 4% is due to the resolution error of the analysed image sequences. In successive stages, the software will be extended to new possibilities of measuring newly discovered features. The selected parts of analysis (main window) are shown in Figure 5.

### Conclusions

The presented software is only one of the possible solutions to the analysis of data from the Corvis tonometer. Particular attention should be drawn here to the piece of software related to the corneal contour detection. This algorithm is implemented in the function *Corvis_read_fun.m*. It involves detection of the largest brightness gradient for successive image matrix columns.

In most cases, the largest brightness changes occur on the outer and inner edges of the cornea. Also other methods of edge detection can be applied here, for example, the Canny method or Roberts or Prewitt filters [26,27]. However, proper interpretation of the detected edge still remains to be resolved. It is a key issue in the search for the proper waveform of the deforming cornea. Approximation of the cornea with a polynomial of degree 3 or 4 not only can introduce false non-existing contour sections but also smooth excessively its waveform. It is also possible to use here other methods known from other imaging areas [28-30]. Readers, by modifying the source code according to their needs, will have the opportunity to carry out this type of research independently. The software can also be extended to the study of a group of patients, database and statistical analysis of a group of patients. Finally, a classifier enabling the classification of specific groups of patients can be proposed [31-34].

The software can be successfully treated as the basis for analysis of corneal deformation and eyeball response images for the Corvis tonometer. In its current form, the software has the following advantages: (1) full automation of measurement; (2) repeatability of the results obtained;(3) lack of licensing restrictions, and (4) possibility of compilation and program operation beyond the Matlab environment (please see Additional file 2 - GUI.avi).

## Availability and requirements

**Project name:** Corvis new parameters

**Project home page:** not available

**Operating system(s):** Windows, Linux

**Programming language:** Matlab R2010b

**Other requirements:** Matlab, Image Processing Toolbox

**Any restrictions to use by non-academics:** no restrictions

The author does not take any responsibility for the consequences of any malfunction of the software.

## Additional files

Additional file 1:
**GUI_Corvis.zip - archive containing 5 m-files.**
Additional file 2:
**GUI.avi - video demonstrating application capabilities (for artificial image).**

## Abbreviation

GUI: Graphical User Interface
